# Lipodystrophy Syndromes: One Name but Many Diseases Highlighting the Importance of Adipose Tissue in Metabolism

**DOI:** 10.1007/s11892-025-01602-5

**Published:** 2025-08-21

**Authors:** Maria Foss-Freitas, Donatella Gilio, Elif A. Oral

**Affiliations:** 1 Metabolism, Endocrinology and Diabetes (MEND) Division, Internal Medicine Department, University of Michigan, 2800 Plymouth Road Building 25, Room 3696, Ann Arbor, MI 48105 USA; 2 Department of Clinical and Translational Sciences, University of Pisa, Pisa, Italy

**Keywords:** Lipodystrophy, Lipoatrophic diabetes, Genotype-phenotype, Adipose tissue, Metabolic abnormalities, Atypical diabetes

## Abstract

**Purpose of Review:**

This review aims to introduce the latest developments in etiology and classification of lipodystrophy syndromes.

**Recent Findings:**

Recent developments in genetic assessment with deeper sequencing have increased the number of specific etiologies of lipodystrophy with known single-gene associations. Despite this, more than 50% of patients diagnosed with partial and most of acquired lipodystrophy do not have a precise disease mechanism. Regardless of the cause of lipodystrophy, patients present with multiple important comorbidities. Complications impact not only metabolic endpoints but the entire body, akin to what happens in extreme obesity.

**Summary:**

As research advances, new subtypes of lipodystrophy are being identified, with recent studies shifting focus from adipocyte differentiation to the role of cellular structures, survival pathways, and immune regulation in the disease etiology. These metabolic diseases pose significant clinical challenges, underscoring the need for further research to understand the mechanisms more precisely, identify new subtypes, and develop targeted therapies.

## Introduction

Lipodystrophy syndromes represent a heterogeneous group of very rare disorders characterized by variable loss of subcutaneous adipose tissue (SAT). Lipodystrophies are classified as either congenital or acquired based on their etiology. Congenital forms are genetically determined and can exhibit either autosomal recessive or autosomal dominant inheritance patterns. Acquired forms are thought to result from autoimmune mechanisms or may be secondary to certain medications, such as highly active antiretroviral therapy (HAART) used in HIV-positive patients. Lipodystrophy syndromes are also categorized as generalized or partial, depending on the extent of subcutaneous fat loss. The four main clinical forms of lipodystrophy include: congenital generalized lipodystrophy (CGL), acquired generalized lipodystrophy (AGL), familial partial lipodystrophy (FPLD) and acquired partial lipodystrophy (APL). Over time, the classification of lipodystrophy syndromes has become increasingly complex, as new phenotypes and disease subtypes have been identified [1]. These disorders are associated with various insulin resistance-related metabolic abnormalities, including atypical diabetes, severe hypertriglyceridemia, and liver fat accumulation that may progress to metabolic dysfunction-associated steatotic liver disease (MASLD) and, in some cases, cirrhosis [2, 3]. Individuals with lipodystrophy face an increased risk of complications such as recurrent acute pancreatitis, proteinuria, renal insufficiency, and cardiovascular disease [4, 5]. Given the progressive nature of these complications, young patients may initially show no metabolic disturbances [6–9].

The specific genetic cause of lipodystrophy can influence clinical signs and symptoms, thereby providing clues for forming a diagnostic suspicion and aiding healthcare practitioners in selecting appropriate genetic tests and facilitating timely diagnoses [10]. Advances in our understanding of adipocyte biology, improved techniques for examining adipocyte function, and progress in genetic sequencing have helped to elucidate the etiology of genetic lipodystrophy syndromes [11], identifying key genes involved in adipocyte differentiation, lipid metabolism, and insulin signaling [12]. Notably, the discovery of nuclear envelope proteins, such as Lamin A and C, as causative factors in lipodystrophy underscores the complexity of these conditions [13–15]. The precise mechanism by which anomalies in these proteins result in selective, time- and location-specific fat loss, along with other pathological changes, remain largely unclear. Additionally, our understanding of the crucial interactions between the immune system and adipocytes, vital for maintaining adipose tissue (AT) function, integrity, and adaptability, is still evolving [16, 17]. As these interconnections become better understood, we anticipate significant breakthroughs in lipodystrophy treatment. This review aims to present the known and proposed disease mechanisms and highlight common themes.

### Generalized Lipodystrophy

Generalized lipodystrophy syndromes (GL) are characterized by a significant reduction or near-total absence of SAT throughout most of the body [18]. In females, the limited presence of SAT emphasizes the appearance of muscular hypertrophy, facilitating earlier diagnosis [2, 19, 20]. In contrast, muscle hypertrophy in males may not be immediately recognized as a symptom of an underlying disorder, often leading to delays in diagnosis [19, 20]. As a result, GL remains frequently underrecognized and underdiagnosed. GL encompass both congenital and acquired forms, which are described individually in the following sections.

#### Congenital Generalized Lipodystrophy

**Fig. 1:**
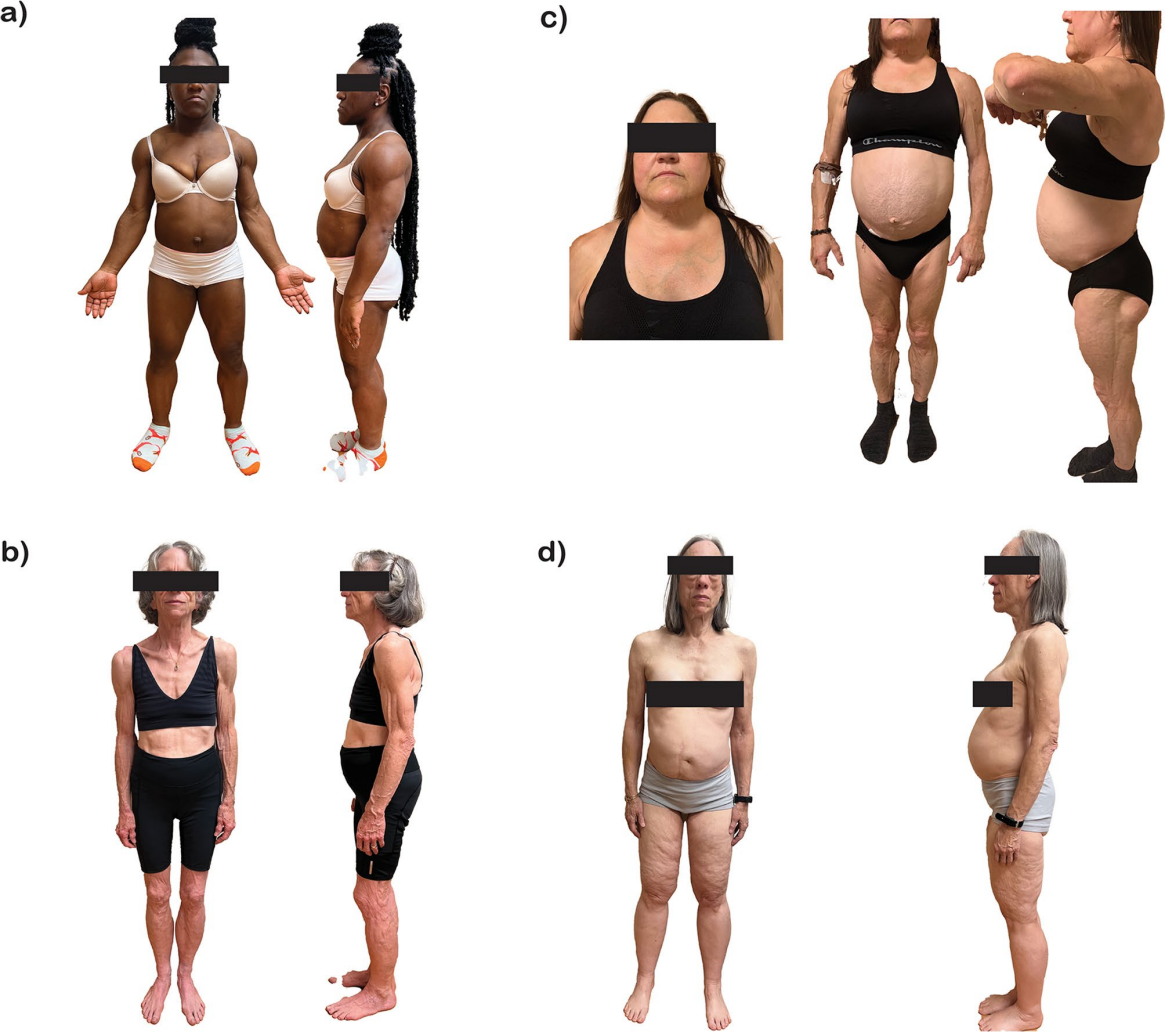
Four broad categories of Lipodystrophy Syndromes (**a**) Congenital Generalized Lipodystrophy (also known as Seip Berardinelli Syndrome). Note generalized fat loss and pseudo-acremegaloid features. (**b**) Acquired Generalized Lipodystrophy (also known as Lawrence syndrome). Even though classically known as a disease of childhood, can present in late adulthood as in the patient presented. (**c**) Familial Partial Lipodystrophy (also known as Köbberling-Dunnigan Syndrome). Note preservation of fat around face and neck. (**d**) Acquired Partial Lipodystrophy (one subtype known as Barraquer-Simons syndrome or cephalocaudal syndrome is presented here). Note preservation of fat below inguinal line

Congenital generalized lipodystrophy, or Berardinelli-Seip Syndrome (OMIM #608594, ORPHA:528), is a severe autosomal recessive disorder, characterized by the near-total absence of SAT, typically evident at birth or within the first year of life (Fig. 1a). First described by Waldemar Berardinelli in 1954, and further detailed by Seip [21, 22], CGL has an estimated prevalence of 1 in 10 million live births, with higher incidence rates in certain regions [23]. While there is no gender predominance, females are diagnosed more frequently, likely due to their greater tendency to seek medical attention for pronounced muscular hypertrophy [1] as well as earlier and more severe metabolic abnormalities. CGL is associated with premature death, reducing patients’ lifespan by 30 years or more, primarily due to liver disease and infections, although the mechanisms underlying susceptibility to infections remain unclear [19]. There are four main subtypes of CGL (types 1 to 4) caused by pathogenic variants (PVs) in genes such as *AGPAT2*, *BSCL2*, *CAV1*, and *PTRF*, respectively. Two additional types are associated with biallelic variants in the *PPARG* and *LMNA* genes. Types 1 and 2 are the most common CGL, accounting for approximately 95% of cases with a known genetic cause (Table 1).

**Table 1:** Characterization of congenital generalized lipodystrophy syndromes and familial partial lipodystrophy syndromes

Syndrome	Inheritance	Gene	Fat distribution	Distinguishing features	Age of onset	Main comorbidities
CGL1	AR	*AGPAT2*	Near-complete absence of SAT, retained mechanical AT	• Accelerated childhood growth • Acromegalic features • Prominent veins • Hypermuscular appearance • Umbilical prominence • Hypoleptinemia	At birth or during the first year of life	• Severe insulin resistance • Early-onset diabetes • HyperTG • Pancreatitis • Hepatic steatosis • Hyperphagia • Hypertrophic cardiomyopathy • Bone cysts • Rare intellectual deficiency
CGL2	AR	*BSCL2*	Near-complete absence of SAT	• Accelerated childhood growth • Acromegalic features • Prominent veins • Hypermuscular appearance • Umbilical prominence • Hypoleptinemia	At birth or during the first year of life	• Severe insulin resistance • Early-onset diabetes • HyperTG • Pancreatitis • Hepatic steatosis • Hyperphagia • Hypertrophic cardiomyopathy • Intellectual deficiency • Rare neurological sign (encephalopathy, spasticity)
CGL3	AR	*CAV1*	Near-complete absence of SAT, preserved bone marrow fat	• Short stature • Premature aging • Prominent veins • Hypermuscular appearance • Hypoleptinemia	At birth or during childhood	• Severe insulin resistance • Dyslipidemia • Hepatic steatosis • Pulmonary arterial hypertension • Megaesophagus • Vitamin D resistance
CGL4	AR	*PTRF (CAVIN1)*	Progressive generalized loss of SAT	• Prominent veins • Hypermuscular appearance • Acromegalic feature • Umbilical prominence • Hypoleptinemia	At birth, childhood, or adulthood	• Dyslipidemia • Hepatic steatosis • Cardiac arrhythmias • Skeletal abnormalities (atlantoaxial instability, lumbosacral scoliosis) • Gastrointestinal dysmotility • Myopathy
PPARG-related CGL	AR	*PPARG*	Progressive generalized loss of SAT	• Prominent veins • Hypermuscular appearance	Childhood	• HyperTG • Pancreatitis • Refractory diabetes • Irregular menstruation • Renal failure
LMNA-related CGL	AD	*LMNA*	Progressive generalized loss of SAT	• Prominent veins • Hypermuscular appearance	Childhood	• HyperTG • Pancreatitis • Diabetes • Hypertension • Liver abnormalities
FPLD1 (Köbberling syndrome)	Polygenic	Unknown	Loss of fat in the lower limbs and gluteo-femoral region. Accumulation of abdominal fat	• Acanthosis nigricans • Hirsutism	Childhood, puberty or adulthood	• Insulin resistance and metabolic syndrome • PCOS
FPLD2 (Dunnigan syndrome)	AD	*LMNA*	Loss of fat in the limbs, trunk and gluteal region. Fat accumulation in the face, neck, axillae, interscapular area, labia majora and abdominal viscera	• Prominent veins • Hypermuscular appearance • Lipomas • Hirsutism	Puberty in women, later in men	• Insulin resistance • Diabetes • HyperTG • Pancreatitis • Hepatic steatosis • PCOS • Fertility problems • Cardiac arrhythmias • Possible association with cardiomyopathy • Skeletal and muscular dystrophy
FPLD3	AD	*PPARG*	Moderate loss of fat in the limbs and gluteo-femoral region. Accumulation of fat in the face and neck may not be present	• Absence of prominent veins • Mild prominent musculature in the forearms and calves	Adolescence or early adulthood	Earlier and severe metabolic complications such as: • Diabetes • Hyperlipidemia • Pancreatitis • Severe hypertension • PCOS
FPLD4	AD	*PLIN1*	Loss of fat in the limbs and gluteo-femoral region. Facial fat accumulation	• Possible presence of muscular hypertrophy • Acromegalic features	Childhood or adulthood	• Severe insulin resistance • Early-onset diabetes with ketosis • HyperTG • Pancreatitis • Hepatic steatosis • Ovarian dysfunction
FPLD5	AR	*CIDEC*	Loss of fat in the limbs and gluteo-femoral region	• Muscular hypertrophy	Childhood	• Severe insulin resistance • Early-onset diabetes • Ketosis • HyperTG • Pancreatitis • Hypertension • Hepatic steatosis
FPLD6	AR	*LIPE*	Loss of fat in the buttocks, hips and lower limbs. Accumulation of fat in the neck, supraclavicular area, axillae, back, abdomen and labia majora	• Multiple lipomatosis • Auto-fluorescent drusen-like retinal deposits	Adulthood	• Diabetes • HyperTG • Pancreatitis • Hypertension • Hepatic steatosis • Muscular atrophy with elevated creatine kinase levels
FPLD7	AD	*CAV1*	Loss of fat in the face and upper body	• Progeroid features	At birth	• Congenital cataracts
AKT2-related FPLD	AD	*AKT2*	Loss of SAT from extremities and femoral-gluteal region, preserved or increased abdominal subcutaneous and visceral fat	NA	Adulthood	• Severe insulin-resistance • Diabetes • Hypertension
PCYT1A-related FPLD	AR	*PCYT1A*	Loss of SAT in the limbs and buttocks	• Short stature • Muscular atrophy	Childhood	• Severe insulin resistance • Diabetes • HyperTG • Pancreatitis • Hepatic steatosis
ADRA2A-related FPLD	AD	*ADRA2A*	Loss of SAT in the limbs and trunk. Accumulation of fat in the neck and intra-abdominal region	• Hypermuscular appearance	Adolescence	• Diabetes • Hypertension • Dyslipidemia
MFN2-related FPLD	AR	*MFN2*	Loss of fat in the limbs, forearms and gluteo-femoral region	• Pseudo-lipomatosis in the upper body	Childhood, adolescence or adulthood	• Diabetes • HyperTG • Hepatic steatosis • Axonal polyneuropathy
NOTCH3-related FPLD	AD	*NOTCH3*	Loss of SAT from the upper and lower extremities	• Hypermuscular appearance • Umbilical prominence		• Diabetes • HyperTG • Hepatic steatosis

^a^CGL: congenital generalized lipodystrophy; AD: autosomal dominant; AR: autosomal recessive; SAT: subcutaneous adipose tissue; AT: adipose tissue; HyperTG: Hypertriglyceridemia; FPLD: familial partial lipodystrophy; PCOS: Polycystic ovary syndrome; NA: not available

CGL Type 1 is caused by PVs in the *AGPAT2* gene on chromosome 9q34, which encodes an enzyme crucial for triglyceride synthesis and fat storage in adipocytes. However, patients can clearly synthesize triglycerides as they display hypertriglyceridemia. The pathway for triacyl-glycerol and phospholipid synthesis converge. These PVs may disrupt this complex pathway with an accumulation of either toxic or proinflammatory metabolites, altering intracellular signaling and ultimately causing adipocyte death. A recent study showed that acute targeting of *AGPAT2* with anti-sense oligonucleotides resulted in accumulation of lysophosphatidic acid, which was sufficient to induce inflammation in both adipocytes and liver [24].

In addition to the generalized absence of SAT, patients may exhibit accelerated growth, acromegaloid features, phlebomegaly, and pronounced musculature. Umbilical hernias or protrusions are also characteristic [1, 19]. Common complications include insulin resistance leading to diabetes mellitus (DM), hypertriglyceridemia-induced pancreatitis, and hepatic steatosis, resulting in hepatomegaly. Other prevalent features include cardiovascular diseases, particularly hypertrophic cardiomyopathy [25], hyperandrogenism, and cysts in long bones. In rare cases, intellectual disability has also been described [26]. Patients typically present with hypoadiponectinemia, very low leptin levels, and experience hyperphagia in early childhood, often appearing as fussy infants [26].

CGL Type 2 is caused by PVs in the *BSCL2* gene on chromosome 11q13, which is essential for triglyceride synthesis, lipid droplet fusion, and adipocyte differentiation. This subtype often manifests with more severe multi-system symptoms compared to other forms of CGL, including cognitive deficits, hypospermia, and hypogonadism in males. Metabolic complications also tend to be more severe and appear earlier, despite higher circulating leptin levels [2, 27]. Although triglyceride synthesis in adipocytes is compromised in these patients, serum triglyceride levels are markedly elevated. This paradox is explained by the significant loss of adipose tissue, which results in ectopic fat deposition and increased circulating free fatty acids. The liver, in turn, metabolizes these excess fatty acids into very low-density lipoproteins (VLDL), leading to a substantial increase in serum triglyceride concentrations [26]. Additionally, the bidirectional nature of lipid metabolic pathways, along with the diversion of lipids into phospholipid synthesis, particularly phosphatidylethanolamine, may contribute to adipocyte loss by inducing lipotoxic stress in precursor cells [28]. Within CGL Type 2, a specific condition known as Celia’s encephalopathy, or Progressive Encephalopathy with or without Lipodystrophy (PELD), is associated with a PV in exon 7 of the *BSCL2* gene, which leads to an aberrant splicing site, resulting in the skipping of exon 7 [29]. In addition to the typical features of CGL2, patients with this PELD experience delays in developmental milestones following birth. Over time, they may develop gait disturbances, along with a loss of speech and comprehension. As the disease progresses, epileptic symptoms and severe encephalopathy can occur. Premature death often results from respiratory sepsis associated with bronchial aspiration or from status epilepticus. Notably, post-mortem studies of two affected patients have revealed significant atrophy of the caudate nuclei [29, 30].

CGL Type 3 is the rarest subtype of congenital generalized lipodystrophy, with only four cases reported to date. It is caused by PVs in the *CAV1* gene on chromosome 7q31. Achalasia has been reported in two of the described cases, leading to dysphagia and progressive megaesophagus, features that may help distinguish this subtype from other forms of CGL [31, 32].

CGL Type 4, documented in just over 40 cases, is caused by PVs in *PTRF* gene. It encodes for a cytoplasmatic protein called caveolae-associated protein 1 (Cavin-1), which, together with caveolin 1, is crucial for the biogenesis of caveolae and regulates the expandability of AT [33]. Cavin-1 is expressed in various tissues, including muscle, leading to specific clinical features in affected individuals. They typically exhibit a near-complete absence of AT accompanied by muscular dystrophy, characterized by elevated creatine phosphokinase (CPK) levels and muscle weakness. Furthermore, patients often present with cardiac arrhythmias, skeletal abnormalities, and gastrointestinal dysmotility, which are highly indicative of this syndrome [34–36].

#### Acquired Generalized Lipodystrophy

Acquired generalized lipodystrophy, also known as Lawrence Syndrome (ORPHA:79086), is characterized by fat loss that can be gradual or associated with panniculitis, typically occurring during childhood or adolescence, with a female predominance [37] (Fig. 1b). Initially, the loss of SAT may be localized to specific areas; however, as the disease progresses, over weeks, months, or, in some cases, years, total body fat can diminish to levels comparable to those seen in CGL. Notably, bone marrow, retro-orbital, and intra-abdominal fat may be spared. Common comorbidities include insulin-resistant DM, hypertriglyceridemia leading to pancreatitis, and hepatic steatosis [37, 38]. Although the exact pathogenesis of AGL remains incompletely understood, an autoimmune etiology is strongly supported by its frequent association with other autoimmune conditions, such as juvenile dermatomyositis (JDM), autoimmune thyroid disease, and systemic lupus erythematosus, pointing toward a mechanism of immune-mediated adipocyte destruction [39]. Furthermore, some AGL patients exhibit activation of the classical complement pathway reflected by low complement C4\ levels [40]. Recent studies have identified autoantibodies targeting the lipid droplet protein perilipin 1 (PLIN1) [41, 42], with their titer significantly correlating with the extent of fat loss, metabolic control impairment, and severity of liver injury [43]. Other specific target antigens, however, have yet to be identified. While autoimmunity is a hallmark in many AGL cases, there may be rare forms of the disease where a clear autoimmune cause is not evident, and further mechanistic studies are needed to explore this. Beyond classical autoimmune associations, AGL has also been linked to various immune system disorders, including common variable immunodeficiency [44], immune thrombocytopenia [45] and coexisting CD28 and CTLA4 haploinsufficiency [46]. Interestingly, there is growing evidence that AGL can be triggered by immune checkpoint inhibitors such as anti-programmed cell death protein 1 (PD-1) therapies, including pembrolizumab and nivolumab, used to treat metastatic melanoma and renal cell carcinoma [47–51]. These observations not only reinforce the autoimmune nature of the disease but also implicate broader immune dysregulation as a potential driver of adipose tissue loss.

Historically, AGL was classified into three subtypes: panniculitis-related (Type 1), autoimmune disease-related (Type 2), and idiopathic (Type 3), each characterized by distinct patterns of SAT loss [38]. Despite this classification, the overlap between these categories is common [38, 52]. A notable association exists between AGL and juvenile dermatomyositis, with approximately 10% of JDM cases developing generalized fat loss [53]. For example, some AGL patients who have been classified in this category concurrently exhibit progeroid features due to a specific pathogenic variant in the *LMNA* gene (p.T10I) [54]. Intriguingly, one such patient also had biopsy-confirmed JDM, suggesting a potential molecular link between these disorders [55]. Nevertheless, it is important to emphasize that *LMNA* mutations have been identified in a minority of AGL cases; the majority do not have a defined genetic cause, underscoring the acquired, rather than inherited, nature of most forms of the disease. As research advances in the molecular mechanisms of inflammatory and immune-related diseases, it is expected that the AGL phenotype will be redefined and reclassified [46, 56].

### Partial Lipodystrophy

Partial Lipodystrophy (PLD) encompasses various metabolic disorders with significant clinical heterogeneity [57, 58]. Even among individuals with identical gene variants, this variability poses challenges for clinical suspicion and accurate diagnosis [18, 58]. A thorough physical examination is vital in the diagnostic process. A hallmark of PLD is the scarcity of SAT, particularly in the extremities, with pronounced loss in the lower limbs and the gluteo-femoral region. However, fat deposition can vary across other body regions, including the face, neck, upper back, intra-abdominal, axillary, and pubic regions.

#### Familial Partial Lipodystrophy

Familial partial lipodystrophy is characterized by the loss of SAT from the upper and lower extremities, with varying degrees of fat loss from the trunk (Fig. 1c**).** While the genetic inheritance pattern of FPLD type 1 remains unclear and may be polygenic [59], the other subtypes are monogenic and are generally referred to by subtype number. FPLD2 is associated with pathogenic variants in *LMNA* (lamin A/C), FPLD3 in *PPARG* (peroxisome proliferator-activated receptor gamma), FPLD4 in *PLIN1* (perilipin 1), FPLD5 in *CIDEC* (DFFA-induced cell death effector C), FPLD6 in *LIPE* (hormone-sensitive lipase), and FPLD7 in *CAV1* (caveolin 1). The field is currently working toward a harmonized gene-based nomenclature to clarify conceptual relationships, moving away from purely numerical classifications. This effort is ongoing, and the manuscript can be updated accordingly once a consensus is reached. These genes play fundamental roles in adipogenesis, contributing to the understanding of the complexity of these disorders [7, 60]. Some FPLD subtypes can present with fat accumulation in the face and supraclavicular region, leading to a Cushingoid appearance with features such as a “buffalo” or “dorsocervical” hump and double chin. For an accurate differential diagnosis, it is essential to recognize that while PLD presents with reduced SAT and normal or hypertrophic muscle, Cushing’s syndrome involves preserved SAT and reduced muscle mass. Although these phenotypic similarities exist, the underlying pathogenic mechanisms differ between FPLD and Cushing’s syndrome. In hypercortisolemia, excess cortisol induces fat redistribution by directly binding to glucocorticoid receptors on adipocytes, which promotes the accumulation of visceral fat and preferential fat deposition in areas such as the face, trunk, and dorsocervical region. Simultaneously, cortisol stimulates lipolysis in subcutaneous adipose tissue, resulting in an increased release of free fatty acids (FFAs) into the bloodstream. These changes are further compounded by muscle wasting, as cortisol exerts catabolic effects on protein metabolism, leading to reduced muscle mass [61]. Conversely, in FPLD, genetic mutations impair adipocyte function and survival, causing selective loss of SAT in limbs and gluteal regions, with relative preservation or hypertrophy of fat depots in areas like the face and neck [61]. This redistribution mimics the Cushingoid phenotype but occurs independently of cortisol excess. The age of onset for the FPLD phenotype usually varies by subtype: FPLD1 typically manifests in the third or fourth decade, FPLD2 during or even before puberty, FPLD3 and FPLD6 in early adulthood, while FPLD4 and FPLD5 may present in childhood [18, 57].

The genetic and clinical diversity of FPLD presents a fascinating and multifaceted challenge, offering valuable insights into the molecular mechanisms underlying metabolic diseases and paving the way for the development of more precise, targeted therapeutic interventions [62].

FPLD1 (Köbberling Syndrome) represents the most common subtype with a seemingly polygenic etiology. Its phenotype is highly variable but typically involves reduced AT in the glutes and lower limbs, coupled with abdominal fat accumulation. In some cases, fat loss extends to the upper limbs, with a characteristic fat distribution that follows a proximal-to-distal gradient, resembling an exaggerated “apple-shaped” body type. Due to its clinical heterogeneity, the prevalence of FPLD1 may vary depending on how the phenotype is defined, and it is likely underdiagnosed. A genome-wide association study identified five distinct clusters of type 2 DM (T2DM) loci and traits, one of which showed a “lipodystrophy-like” fat distribution. This finding has significant implications for the pathogenesis of T2DM, a condition that affects approximately 537 million people worldwide, as reported by the International Diabetes Federation (IDF) in 2021. Notably, about 90% of these cases are classified as T2DM. In a more recent and larger dataset analysis, some of the same authors identified two clusters out of a total of 12 within the broader Type 2 diabetes population, encompassing a more diverse multi-ethnic cohort. These two clusters exhibit features resembling some form of lipodystrophy, where altered fat distribution appears to be linked to the development and presentation of Type 2 diabetes through specific mechanisms [63] Given these observations, it is possible that some T2DM cases, particularly those resembling FPLD1, may represent undiagnosed cases of familial partial lipodystrophy [64].

FPLD 2 (Dunnigan Syndrome) is the most prevalent and well-characterized monogenic form of FPLD. This variant was first described by Dunnigan in 1974 and later corroborated by Köbberling in 1975. Individuals affected by FPLD2 are born with a normal fat distribution but gradually develop a progressive loss of AT in the extremities, trunk, and gluteal region. Simultaneously, abnormal fat accumulation occurs in the neck, face, and intra-abdominal region, leading to a cushingoid appearance. The precise onset of body fat changes and metabolic complications during childhood remains unclear. Marked phenotypic heterogeneity has been reported in children affected by FPLD2, particularly among girls, some of whom exhibit FPLD2 symptoms and metabolic complications, such as hypertriglyceridemia, even before puberty [65]. These observations suggest that the adipose phenotype may develop before the onset of puberty. They also raise interesting questions regarding sex-specific differences in disease presentation and severity. Studies indicate that men tend to have a less severe metabolic profile compared to women, who show a higher prevalence of complications such as DM, atherosclerotic cardiovascular disease, hypertriglyceridemia, and low high-density lipoprotein cholesterol (HDL-C) levels [66, 67].

The FPLD2 is more readily recognized in women than men due to the unusual muscular appearance of the extremities, the higher prevalence of hirsutism, menstrual irregularities, polycystic ovary syndrome (PCOS), and earlier, more severe metabolic manifestations. Fat accumulation in the genital region is commonly observed in female patients, and our clinical findings suggest that men may also exhibit fat deposition in this area, although it has been less frequently reported in the literature. However, this has been less frequently noted. Additionally, some patients may experience myopathy, cardiomyopathy, and cardiac electrical conduction disorders, supporting the concept of a “multisystem dystrophic syndrome” [68–70].

The genetic basis of FPLD2 is well established and linked to PVs in *LMNA* gene (1q21-22). The lamins A and C, encoded by *LMNA*, are crucial components of the nuclear lamina, located between the inner nuclear membrane and chromatin. They are involved in nuclear organization, chromatin assembly, nuclear membrane integrity, and telomere stability, with a marked presence in the cytosol, cytoskeleton, and cell nucleus [71]. Mutations in *LMNA* likely impair nuclear function, leading to delipidation and early adipocyte apoptosis or death. Approximately 75% of FPLD2 cases present a pathogenic variant in exon 8, where arginine at position 482 is replaced by a neutral amino acid such as glutamine, leucine, or tryptophan. Other variants in exon 9 and 11 have also been identified. The R482W variant is most strongly associated with muscular and cardiac abnormalities, including muscular atrophy, cardiac hypertrophy and development of atrial fibrillation, and advanced atherosclerosis. In rare cases, PVs in exon 1 cause cardiomyopathy, early-onset congestive heart failure, and cardiac arrhythmias, sometimes necessitating heart transplantation. However, further research is needed to fully understand the phenotypic differences associated with each specific variant causing FPLD2 [72]. FPLD2 is now part of a broader group of genetic conditions known as laminopathies, which are caused by changes in the nuclear lamina. Other laminopathies include Hutchinson-Gilford progeria, mandibuloacral dysplasia, Emery-Dreyfuss muscular dystrophy, pelvic and scapular girdle muscular dystrophy type 1B, type 1 A dilated cardiomyopathy, and Charcot-Marie-Tooth disease, among others [73].

FPLD3 is caused by PVs in the *PPARG* gene located on chromosome 3 (3p25). This gene encodes the PPARγ receptor (peroxisome proliferator-activated receptor gamma), a nuclear transcription factor that belongs to the nuclear receptor family. PPARγ is primarily expressed in AT and plays a pivotal role in adipocyte differentiation. The receptor forms a heterodimer with the retinoic-acid X-receptor (RXR) and, when activated by ligands, it triggers a cascade of events that lead to systemic insulin sensitization. In AT, this leads to increased lipid uptake and storage, expansion in the adipocyte number, and recruitment of activated macrophages. In skeletal muscle, PPARγ activation enhances insulin sensitivity, while in the liver it suppresses gluconeogenesis [74]. Pathogenic variants impairing the *PPARG* function result in reduced receptor activity, leading to a lipoatrophic phenotype [75]. Interestingly, there is an in vitro study that classifies all possible missense mutations in *PPARG* gene through a functional assay that can add in the likelihood of attribution of pathogenicity if a missense variant is observed [76]. Compared to individuals with FPLD2, those with FPLD3 exhibit milder fat loss, affecting predominantly the distal extremities, especially the calves and forearms. In contrast, fat in the thighs and upper arms is relatively preserved. Despite this, FPLD3 patients often experience more severe metabolic complications, suggesting that the remaining AT may be dysfunctional or that individuals with milder metabolic disease may be underdiagnosed [77]. Approximately 30 cases of FPLD3 have been thoroughly described in the literature, highlighting its rarity and clinical complexity and the need for deeper understanding to ensure accurate diagnosis and management [1, 18, 76].

FPLD4 is caused by PVs in the *PLIN1* gene, located on chromosome 15 (15q26). This gene encodes perilipin 1, the most abundant protein coating adipocyte lipid droplets. Perilipin 1 plays a crucial role in the formation and maturation of these droplets, regulating triglyceride storage and the release of free fatty acids [78]. Defects in perilipin 1 lead to constitutive activation of triglyceride lipase in AT, causing elevated basal lipolysis. This dysfunction also reduces adipocyte size, induces AT fibrosis, and increases macrophage infiltration, creating an inflammatory profile that exacerbates metabolic issues [78, 79]. Only six cases of FPLD4, documented in three families, have been described in the literature. Affected patients presented with hyperinsulinemia, hypertriglyceridemia, and hepatic steatosis, along with more pronounced lipodystrophy in the lower limbs and gluteo-femoral regions [18, 57]. Recent studies suggest that *PLIN1* haploinsufficiency may not always cause lipodystrophy and could even confer a favorable metabolic profile, potentially protecting against cardiovascular disease [80, 81]. This challenges the earlier belief that PLIN1 haploinsufficiency is pathogenic. Instead, it appears that only specific frameshift variants, which extend the translated protein, lead to severe lipodystrophy, likely through a dominant-negative mechanism [80]. These data highlight that the pathogenicity of *PLIN1* mutations is complex and may depend on the precise nature of the mutation rather than a simple loss of function [80].

FPLD5 and FPLD6 are caused by recessive mutations in cell death-inducing DFFA-like effector C (*CIDEC*) and Hormone-Sensitive Lipase (*LIPE*) genes, respectively [82]. A nonsense variant in the *CIDEC* gene has been associated with severe insulin resistance and acanthosis nigricans beginning in childhood [83]. In contrast, FPLD6 typically presents in adulthood and is characterized by progressive symmetric myopathy. This condition can also lead to fat accumulation in the neck, back, and supraclavicular areas, which may resemble the dorsocervical fat accumulation seen in Cushing syndrome [84].

FPLD7 is due to variants in the *CAV1*. Patients with this syndrome presented neonatal onset lipodystrophy, with loss of fat in the face and upper body, as well as certain progeroid features, the presence of cataracts and dyslipidemia [85, 86].

Recently, a novel subtype of FPLD caused by gain-of-function missense variants in the negative regulatory region of the *NOTCH3* gene has been described [87]. In addition to these subtypes, there are patients with a phenotype and family history suggestive of FPLD who lack known gene variants, suggesting the existence of other genetic loci that have not yet been investigated. Table 1 provides a detailed summary of all FPLD subtypes and linked genetic defects.

#### Acquired Partial Lipodystrophy

Historically, acquired partial lipodystrophy primarily referred to Barraquer-Simons syndrome, a rare condition usually presenting in childhood or adolescence, marked by a characteristic, region-specific loss of fat. However, other APL forms have been identified and added to the classification, including those related to HIV infection. Below is an overview of the main subtypes.

The most prevalent form of APL affects approximately 50% of individuals with human immunodeficiency virus (HIV) and has been associated with the use of earlier highly active antiretrovirals, including first- and second-generation protease inhibitors (PIs) [88]. First-generation PIs, such as nelfinavir and indinavir, negatively impact AT by inhibiting adipocyte differentiation, promoting insulin resistance, and increasing the production of pro-inflammatory cytokines by adipocytes and macrophages infiltrating the tissue [89]. Second-generation PIs, such as ritonavir and lopinavir, further damage AT by inducing oxidative stress and altering adipokine secretion patterns [89, 90]. Regarding nucleoside analog reverse transcriptase inhibitors (NRTIs), first-generation molecules like stavudine and zidovudine contribute to subcutaneous lipoatrophy, while second-generation NRTIs, such as tenofovir, do not exhibit these effects [91]. Beyond the impact of antiretroviral drugs, HIV infection itself can contribute to the development of lipodystrophy. Infection of adipocytes and macrophages, which act as reservoirs of viral replication, promotes the release of viral proteins that disrupt the adipocyte phenotype. Additionally, macrophages transition from an anti-inflammatory (M2) to a pro-inflammatory (M1) pattern leads to the production of pro-inflammatory cytokines, which further aggravate insulin resistance and contribute to AT dysfunction [92]. In individuals with HIV, APL is characterized by a redistribution of body fat, with notable fat loss in the upper and lower limbs, the femoral-gluteal region, and the face [93]. Conversely, fat accumulation occurs primarily in the visceral abdominal area, mammary tissue in women (less frequently in men), and occasionally in the dorsocervical area [94]. These alterations, collectively termed HIV-associated lipodystrophy or HIV metabolic syndrome, often present alongside an atherogenic profile, increasing the risk of cardiovascular diseases [95, 96] and seem to be correlated to age, gender, and HIV infection duration [97]. In such patients, serum leptin levels are typically normal or elevated, whereas adiponectin levels are reduced [98].

The best-known form of APL not HIV-related is Barraquer-Simons Syndrome (or cephalocaudal lipodystrophy, OMIM #608709, ORPHA:79087). It is characterized by a progressive loss of SAT in the upper body, with a symmetrical and cephalocaudal progression (Fig. 1d). It predominantly affects females and generally begins in childhood or adolescence, impacting the face, neck, shoulders, arms, and trunk while sparing the abdomen and lower limbs. As a result, there is often an excessive accumulation of AT in the lower body [1, 99]. Although metabolic complications are uncommon, likely due to the preserved lower body fat [100], some patients with more extensive fat loss and longer disease course have reported severe metabolic manifestations. In such cases, increased inflammation and fibrosis in the lower-body fat may contribute to these metabolic disturbances, highlighting the need for close monitoring to address any emerging metabolic alterations [8, 9].

To date, approximately 250 cases of Barraquer-Simons Syndrome have been reported, predominantly among individuals of European descent without a family history of lipodystrophy. Unlike other forms of lipodystrophy, this syndrome does not demonstrate hereditary patterns and is not linked to pathogenic variants in genes typically associated with lipodystrophy. However, some cases have identified variants in the *LMNB2* gene, although the potential genetic contributions to this condition remain unclear [101]. Nonetheless, these findings may suggest a complex trait with an underlying genetic susceptibility.

Several autoimmune diseases, including systemic lupus erythematosus and dermatomyositis, have been reported in conjunction with this condition, with many patients testing positive for at least one of the autoantibodies commonly screened for autoimmune conditions like ANA (antinuclear antibody) and ENA (extractable nuclear antigen) panel tests [100, 102, 103]. Following lipodystrophy onset, about 20–40% of patients develop membranoproliferative glomerulonephritis, and up to 80% exhibit low serum C3 complement levels [104]. The serum concentrations of the classical complement pathway components (C1q, C2, C4) remain within the normal range, suggesting that C3 is activated via the alternative pathway. C3 nephritic factor autoantibody (C3NF), a pool of polyclonal immunoglobulin G, has been found in almost 70% of patients, suggesting that it mediates the activation of the alternative complement pathway by stabilizing the C3 convertase enzymatic complex [103, 105]. The potential mechanisms linking C3 hypocomplementemia, alternative complement activation, and the site-specific reduction of SAT remain unclear, but these findings strongly suggest an autoimmune pathogenesis similar to that of AGL. Infections have also been proposed as potential environmental triggers for autoimmunity, as it has been reported that infections frequently precede the onset of Barraquer-Simons Syndrome, particularly measles [100, 104], varicella, rubella, mumps, and meningitis [106, 107].

In addition to the above, APL may also occur in cancer patients undergoing whole-body radiotherapy, cytotoxic chemotherapy, or in the context of graft versus host disease (GVHD) after hematopoietic stem cell transplantation, particularly when treatment is initiated at a young age (typically < 10 years). In these cases, SAT loss occurs in the limbs and buttocks, while fat is preserved in the face, neck, and abdomen, with more pronounced visceral fat deposition. Such fat redistribution is often accompanied by metabolic complications like insulin resistance, DM, hypertriglyceridemia, and fatty liver disease. While most patients experience partial fat loss, the condition may sometimes progress to more generalized fat loss over time. It has been hypothesized that cytotoxic treatments administered during a critical window for adipose stem cell commitment may impair adipose tissue expandability and regional adipogenesis. In this context, damage to adipose progenitor cells and inflammatory cues may alter adipose tissue remodeling and contribute to the development of APL [108–111].

Furthermore, several autoinflammatory syndromes associated with acquired fat loss, such as joint contractures, muscle atrophy, microcytic anemia, and panniculitis-induced lipodystrophy (JMP) syndrome [112], Japanese autoinflammatory syndrome with lipodystrophy [113] and chronic atypical neutrophilic dermatosis with lipodystrophy and elevated temperature (CANDLE) syndrome [114] have been associated with PVs of the proteasome subunit beta-type 8 (*PSMB8*) gene.

### Atypical (or Unusual) Lipodystrophy

Other genetic lipodystrophy syndromes have been reported but have not been definitively classified. These forms can manifest with diverse fat distribution patterns and metabolic complications and, in some cases, may overlap with other syndromes. Several forms are associated with progeroid traits (Table 2).

**Table 2:** Summary of the main characteristics of the different subtypes of progeroid syndrome associated with lipodystrophy

Syndrome	Inheritance	Gene	Fat Distribution	Distinguishing Features	Main Comorbidities
Werner Syndrome [134]	AR	*RECQL2 (WRN)*	Partial lipodystrophy (mainly affecting the extremities)	• Short stature • Progeroid signs	• Diabetes mellitus • HyperTG • Hypogonadism • Atherosclerosis • Cataracts • Osteoporosis • Strong predisposition to a variety of neoplasms
SHORT - Syndrome short stature, hyperextensibility, ocular depression, Rieger anomaly, and teething delay syndrome [135]	AD	*PIK3R1*	Lipodystrophy mainly affecting the face, upper extremities, buttocks	• Short stature • Progeroid signs • Hyperextensibility of joints • Ocular depression • Rieger anomaly • Teething delay • Facial dysmorphism • Microcephaly	• Insulin resistance • Diabetes mellitus • Pulmonary stenosis • Hearing loss • Delayed bone age
Bloom Syndrome [136]	AR	*RECQL3 (BLM)*	Scant facial fat in childhood, visceral obesity in adulthood	• Progeroid signs • Prenatal and postnatal growth deficiency with short stature • Long and narrow face • Prominent ears • Retrognathia/micrognathia • Malar flattening • Polydactyly and skin abnormalities	• Insulin resistance • Diabetes mellitus • Dyslipidemia • Hypothyroidism • Early onset of neoplasms • Male infertility • Photosensitivity
Fontaine Progeroid Syndrome [137]	AD	*SLC25A24*	Generalized lipodystrophy	• Progeroid signs • Prenatal and postnatal growth deficiency with short stature • Sparse scalp hair • Wrinkled skin with prominent veins • Hypertrichosis • Triangular face with micrognathia • Wide and convex nasal bridge • Microphthalmia • Low-set dysplastic ears	• Conductive hearing impairment • Muscle weakness • Bone abnormalities • No metabolic alteration
HGPS- Hutchinson Gilford progeria syndrome [138]	AD	*LMNA*	Severe generalized lipoatrophy	• Progeroid signs • Growth retardation • Micrognathia • Prominent eyes • Dental crowding • Beaked nose • Alopecia • Skin abnormalities • Contractures and joint stiffness	• Severe atherosclerosis in childhood • Muscular atrophy • Acroosteolysis affecting the distal phalanges and clavicles • Osteopenia
Type A mandibuloacral dysplasia (MADA) [139]	AR	*LMNA*	Lipodystrophy in the extremities	• Progeroid signs • Mandibular hypoplasia • Growth retardation and short stature • Progressive stiffness or contractures of joints • Beaked nose • Skin mottled pigmentation	• Hyperinsulinemia • Low HDL-cholesterol • Bone abnormalities
Type B mandibuloacral dysplasia [140]	AR	*ZMPSTE24*	Generalized lipodystrophy	• Progeroid signs • Mandibular hypoplasia • Short stature • Skin abnormalities	• Insulin resistance • Diabetes mellitus • HyperTG • Bone abnormalities • Focal segmental glomerulosclerosis
Atypical progeroid syndrome [141]	AD	*LMNA*	Generalized or partial lipodystrophy	• Progeroid signs • Micrognathia • Prominent eyes • Dental crowding • Baked nose • Skin abnormalities • Contracture and joint stiffness	• Severe metabolic complications in early life • Cardiovascular disorders such as dilated cardiomyopathy, coronary artery disease, valvular disease, arrhythmias • Sensorineural deafness • Osteoporosis
Néstor-Guillermo progeroid syndrome [142]	AR	*BANF1*	Generalized lipodystrophy	• Progeroid signs • Growth retardation • Skin abnormalities • Sparse hair on the scalp • Small retrognathic chin • Prominent eyes • Dental crowding • Convex nasal ridge	• Bone abnormalities • Cardiovascular disorders (moderate tricuspid insufficiency, severe mitral regurgitation, pulmonary hypertension) • No relevant metabolic alterations
MDPL- mandibular hypoplasia, deafness, progeroid features, and lipodystrophy syndrome [143]	AD	*POLD1*	Generalized lipodystrophy with increased visceral fat	• Progeroid signs • Growth retardation • Mandibular hypoplasia • Stiff joints • Prominent eyes • Narrow mouth with dental crowding • Beaked nose with bird-like facies • Skin abnormalities	• Insulin resistance • Diabetes • Dyslipidemia • Hepatic steatosis • Muscle atrophy • Sensorineural hearing loss • Hypogonadism in males
Marfanoid-progeroid-lipodystrophy syndrome (MFLS) [144]	AD	*FBN1*	Generalized lipodystrophy	• Progeroid signs • Prematurity, accelerate growth with poor weight gain • Arachnodactyly and digital hyperextensibility	• Myopia • Ectopia lentis • Dural ectasia
Cockayne syndrome type A and B [145]	AR	*ERCC6 / ERCC8*	Not specified	• Progeroid signs • Wrinkled skin • Growth impairment	• Severe mental retardation and neurological manifestations due to a progressive demyelination process • Sensorineural hearing loss • Cataracts and retinal dystrophy • Atherosclerosis • Hypertension • Rare metabolic disorders
Keppen-Lubinsky syndrome [146]	AD	*KCNJ6*	Partial or generalized lipodystrophy	• Progeroid signs • Hypertonia and hyperreflexia • Microcephaly • Micrognathia • Prominent eyes • Narrow nasal bridge • Tented upper lip • High palate • Open mouth.	• Intellectual disability • Seizures
Ruijs-Aalfs syndrome [147]	AR	*SPRTN*	Mild lipodystrophy	• Progeroid signs • Development delay • Triangular face • Frontal bossing • Small deep-set eyes • Bulbous nose with high nasal bridge • Micrognathia • Thoracic kyphoscoliosis and pectus excavatum	• Muscle atrophy • Delayed bone aged • Bilateral cataracts • Early onset hepatocellular carcinoma
Wiedemann-Rautenstrauch syndrome [148]	AR	*POLR3A*	Generalized lipodystrophy	• Progeroid signs • Prenatal and postnatal growth deficiency • Sparse scalp hair • Prominent forehead veins • Relative macrocephaly • Triangular face with mandibular hypoplasia • Hypertelorism • Small palpebral fissures • Broad nasal root and pointed nasal tip • Low-set ears • Small mouth • Pointed chin	• Rare cardiac alterations • No metabolic abnormalities
Lipoatrophic diabetes syndrome due to variants in EPHX1 gene [149]	De novo	*EPHX1*	Not specified	• Dysmorphic and progeroid signs	• Hepatic cytolysis, • Sensorineural hearing loss
Penttinen syndrome [150]	AD	*PDGFRB*	Generalized lipodystrophy	• Progeroid signs • Progressive joint contractures • Kyphoscoliosis • Skin abnormalities • Pseudoprognathism • Palpebral malocclusion	• Corneal opacity • Muscle atrophy • No metabolic complications
Warburg-Cinotti syndrome [151]	AD	*DDR2*	Lipoatrophy in face, hands and feet	• Progeroid signs • Thin nose and small alae nasi • Generalized joint enlargement of the fingers, joint swelling and contractures. • Narrow palpebral fissures • Long face • Posteriorly rotated ears • Skin abnormalities	• Corneal vascularization with reduced vision • No metabolic abnormalities

^a^AR: autosomal recessive; HyperTG: Hypertriglyceridemia; AD: autosomal dominant

^b^ for each syndrome, only one representative reference has been included in the table, selected among the most relevant ones, due to reference number limitations. Additional references are available upon request from the authors

Special mention should be given to multiple symmetric lipomatosis (MSL), also known as Madelung Syndrome or Launois-Bensaude disease, first described by Otto Madelung in 1888, without a clearly defined etiology. This rare disorder is marked by symmetrical fat deposition in the neck and upper body, with lipoatrophy in the distal limbs [115]. This abnormal fat accumulation can lead to deformities, restricted joint mobility, significant pain, and, in severe cases, airway compression, causing respiratory failure. It is often associated with metabolic alterations, including insulin resistance, impaired glucose tolerance, fatty liver disease, dyslipidemia, peripheral neuropathy, autonomic neuropathy, elevated lactate, and extremely low leptin and adiponectin levels [116]. Chronic alcohol abuse is a common risk factor for sporadic MSL cases [117]. Additionally, the m.8344 A > G mutation, which is linked to mitochondrial myoclonus epilepsy and ragged red fibers (MERRF) [118–120], as well as mitochondrial deletions have been identified in some individuals with MSL [121].

The association between mitofusin 2 (*MFN2*) mutations and MSL was first identified in a family where three patients carried compound heterozygous pathogenic variants of *MFN2* (p.G108R and p.R707W) [122]. Further case series reported patients with MSL, partial lipodystrophy, and distal axonal neuropathy, all carried biallelic *MFN2* variants (homozygous recessive p.R707W) [116, 122, 123]. The *MFN2* gene is ubiquitously expressed and encodes a GTPase protein localized in the mitochondrial outer membrane and involved in mitochondrial dynamics [124]. Many *MFN2* variants, scattered throughout the gene, have been identified as the cause of Charcot-Marie-Tooth disease type 2 (CMT2A), a hereditary axonal polyneuropathy [125–128]. However, it is noteworthy that all patients with MSL resulting from *MFN2* mutations carry the same pathogenic variant, p.Arg707Trp, either in a homozygous or compound heterozygous state. This variant resides in the carboxy-terminal domain of MFN2, which is essential for mitochondrial membrane fusion and interaction [129, 130]. The rare patients carrying biallelic *MFN2* pathogenic variants other than p.Arg707Trp have never been reported to display lipomatosis [122, 124, 125, 131–133]; however, they typically present with early-onset and often severe CMT2A. Individuals carrying a single *MFN2* p.Arg707Trp mutated allele do not typically develop MSL, though they may show mild signs of CMT [131]. Given the rarity and complexity of MSL, affected individuals should seek consultation from specialists in rare diseases to ensure accurate diagnosis and appropriate treatment planning.

## Conclusions: Unifying Pathophysiology

Lipodystrophy syndromes are a group of diverse diseases sharing the common theme of inadequate fat depots and function leading to metabolic diseases. These syndromes underscore the critical role of AT expandability and function in maintaining metabolic homeostasis. Indeed, outside of pancreatic beta-cell dysfunction, it is only the absence or inadequate function of AT that can drive the development of DM in rodents and humans alike. As our understanding of the key processes involved in AT development and function improves, we gain deeper insights into defining novel lipodystrophy subtypes. While early studies primarily focused on adipocyte differentiation, recent discoveries have emphasized the significance of cellular structural elements and pathways that regulate cell survival in the etiology of lipodystrophy. Furthermore, immune dysfunction and regulation seem to crosstalk with adipocyte function and/or impact the survival of AT, emerging as important causative disease mechanisms of “acquired” lipodystrophy syndromes. It should also be noted that loss of adipose tissue can be programmed due to genetic defects but can manifest later in life after continuous environmental pressures or the genetic program itself. Regardless of the specific cause, the resulting metabolic defects present significant clinical challenges. Continued research is essential to uncover the precise mechanisms involved, identify novel subtypes, and develop more targeted therapies to manage the metabolic complications of lipodystrophy syndromes and potentially “replace” the function of these cells.

### Key References

- Mancioppi V, Daffara T, Romanisio M, Ceccarini G, Pelosini C, Santini F, et al. A new mutation in the *CAVIN1/PTRF* gene in two siblings with congenital generalized lipodystrophy type 4: case reports and review of the literature. Front Endocrinol (Lausanne). 2023;14:1212729. 10.3389/fendo.2023.1212729.

Phenotypic description of two pediatric siblings with congenital generalized lipodystrophy type 4 (CGL4) caused by a novel homozygous mutation in the *CAVIN1/PTRF* gene.

- Akinci G, Alyaarubi S, Patni N, Alhashmi N, Al-Shidhani A, Prodam F, et al. Metabolic and other morbid complications in congenital generalized lipodystrophy type 4. Am J Med Genet A. 2024;194 [6]:e63533. 10.1002/ajmg.a.63533.

This international case study is the largest reporting clinical outcomes in congenital generalized lipodystrophy type 4 (CGL4).

- Mandel-Brehm C, Vazquez SE, Liverman C, Cheng M, Quandt Z, Kung AF, et al. Autoantibodies to Perilipin-1 Define a Subset of Acquired Generalized Lipodystrophy. Diabetes. 2023;72 [1]:59–70. 10.2337/db21-1172.

New insights into the immune response specificity in a subset of idiopathic acquired lipodystrophy cases further support the growing body of evidence suggesting PLIN1 autoantibodies as a potential biomarker for this syndrome.

- Akinci B, von Schnurbein J, Araujo-Vilar D, Wabitsch M, Oral EA. Lipodystrophy Prevalence, “Lipodystrophy-Like Phenotypes,” and Diagnostic Challenges. Diabetes. 2024;73 [7]:1039-42. 10.2337/dbi24-0018.

Familial partial lipodystrophy syndromes and lipodystrophy-like phenotypes share common pathophysiological features and represent a spectrum of adipocyte dysfunction, which complicates diagnosis. Recognizing lipodystrophy-like phenotypes, particularly in diabetes patients, is crucial for tailored risk management and improved care. However, it is vital to distinguish rare lipodystrophy syndromes as distinct conditions to ensure precise and targeted interventions.

- Smith K, Deutsch AJ, McGrail C, Kim H, Hsu S, Huerta-Chagoya A, et al. Multi-ancestry polygenic mechanisms of type 2 diabetes. Nat Med. 2024;30 [4]:1065-74. 10.1038/s41591-024-02865-3.

In a recent analysis of a larger, multi-ethnic Type 2 diabetes dataset, two of 12 identified clusters showed features resembling lipodystrophy. These clusters are characterized by altered fat distribution linked to specific mechanisms underlying Type 2 diabetes development and presentation.

- Vasandani C, Li X, Sekizkardes H, Brown RJ, Garg A. Phenotypic Differences Among Familial Partial Lipodystrophy Due to LMNA or PPARG Variants. J Endocr Soc. 2022;6 [12]:bvac155. 10.1210/jendso/bvac155.

Subjects with Familial partial lipodystrophy (FPLD) type 3 have milder lipodystrophy but experience more severe metabolic complications compared to those with FPLD2. This may indicate that the remaining adipose tissue in FPLD3 is dysfunctional, or that mild metabolic disease is underrecognized. These findings suggest that adipocyte dysfunction, in addition to the loss of adipose tissue, contributes to metabolic abnormalities in lipodystrophy patients.

- Garg A, Xing C, Agarwal AK, Westfall AK, Tomchick DR, Zhang X, et al. Gain of function NOTCH3 variants cause familial partial lipodystrophy due to activation of senescence pathways. Diabetes. 2024. 10.2337/db24-0624.

Description of a novel subtype of Familial partial lipodystrophy (FPLD) caused by gain-of-function missense variants in the negative regulatory region of the *NOTCH3* gene.

## Data Availability

No datasets were generated or analysed during the current study.
